# High serum uric acid levels explains the negative impact of the altitude adaptation index on the brain electroencephalographic Microstate D under high-altitude hypoxic conditions

**DOI:** 10.3389/fnint.2026.1747838

**Published:** 2026-04-23

**Authors:** Jie Xu, Shurong Jia, Yongjun Jing, Hongyan Li

**Affiliations:** 1 Xizang Autonomous Region Key Laboratory for High Altitude Brain Science and Environmental Acclimatization, Xizang University, Lhasa, China; 2 China Railway No.10 Engineering Group Co., Ltd., Shandong, Jinan, China; 3 Xizang Fokind Hospital, Lhasa, China

**Keywords:** cognitive function, electroencephalographic (EEG) microstates, high-altitude adaptation, mediation effect, uric acid

## Abstract

**Objective:**

To explore the mediating role of serum uric acid (SUA) in the relationship between the acclimatization/adaptation index (AAI) and the transition probability of electroencephalographic (EEG) Microstate D within cognitive control networks under hypoxic conditions at 3,650 m.

**Methods:**

A total of 173 high-altitude residents in Lhasa were recruited. Their peripheral oxygen saturation (SpO₂), hematocrit (HCT), and SUA levels were measured, and resting-state EEG data were recorded. AAI was calculated, and the transition probability of Microstate D was analyzed. Correlation analyses and bootstrap methods were employed to examine the mediating effect of SUA.

**Results:**

AAI was significantly negatively correlated with SUA, and the maladaptive group exhibited significantly higher SUA levels. SUA mediated the relationship between AAI and the outward transition probabilities from Microstate D to Microstates A and C, but no significant mediating effect was found for inward transition probabilities.

**Conclusion:**

Under high-altitude hypoxic environments, SUA serves as a key mediator through which AAI modulates the outward transitions of Microstate D. Regulating SUA levels and enhancing AAI may play a crucial role in preserving cognitive control network function in high-altitude populations.

## 1 Introduction

High-altitude environments are characterized by low barometric pressure, low oxygen levels, and arid climate. Among these environmental factors, low barometric pressure and hypoxia exert the most significant impact on the human body (Li et al., 2022). Exposure to hypoxic conditions at high altitudes affects various organ functions. Studies have shown that in high-altitude environments, hypoxia promotes muscle breakdown, accelerates cellular metabolism, and enhances the breakdown of adenosine diphosphate, thereby increasing uric acid production. Meanwhile, lactate accumulation and sympathetic nervous system activation inhibit renal excretion, leading to uric acid retention in the blood and elevated serum uric acid (SUA) levels (Baillie et al., 2007). Moreover, SUA levels increase progressively with rising altitude, demonstrating a positive correlation (Lu et al., 2015).

Due to substantial individual differences, the level of altitude acclimatization varies among individuals. The degree of altitude acclimatization can be quantitatively characterized by the altitude acclimatization/adaptation index (AAI), which is defined as the ratio of peripheral oxygen saturation (SpO₂) to hematocrit (HCT) (i.e., AAI = SpO₂/HCT). This index effectively assesses the acclimatization or adaptation status of both high-altitude migrants and native residents (Li et al., 2024). Furthermore, a study investigating the effect of SUA levels on blood cell parameters has revealed a positive correlation between SUA and HCT (Chen et al., 2015). Based on these findings, it can be inferred that in high-altitude hypoxic environments, SUA levels increase with rising altitude and exert a positive regulatory effect on HCT, thereby potentially reducing an individual’s capacity for altitude acclimatization. This may exacerbate the impact of high-altitude hypoxia on critical organs, particularly the brain, which is highly sensitive to oxygen deprivation.

Although the brain accounts for only approximately 2% of total body weight, it consumes more than 20% of the body’s total oxygen supply, reflecting its extremely high metabolic rate and its critical dependence on a sustained and adequate oxygen supply for normal functioning (Raichle and Gusnard, 2002; Li et al., 2013). As altitude increases, the partial pressure of atmospheric oxygen gradually decreases, leading to a decline in blood oxygen saturation and a reduction in the oxygen available to the brain. This, in turn, disrupts the normal physiological activities of neural cells; moreover, higher altitudes are often associated with more severe cognitive impairment (Meng et al., 2023). The impact of hypoxia on cognitive function is broadly reflected in multiple domains, including visual abilities, memory, attention, and executive function. The prefrontal cortex, parietal lobe, and frontoparietal junction—key brain regions involved in the regulation of active attention allocation, cognitive conflict resolution, and executive control—exhibit high levels of cellular activity and energy demand, rendering them particularly vulnerable to hypoxic conditions. Research has shown that high-altitude hypoxia induces structural damage in these brain regions, including gray matter atrophy and white matter microstructural abnormalities, through mechanisms such as oxidative stress, altered cerebral hemodynamics resulting from polycythemia, and molecular regulation via the HIF-EPO pathway. These changes inhibit the synchronization of neuroelectrophysiological activity in the aforementioned regions, impair the spatial integration and temporal maintenance of neural activity, and ultimately compromise the functions of these areas, leading to physiological and behavioral abnormalities related to cognitive regulation. Consequently, when an individual has a poorer capacity for altitude acclimatization, the impairment of these related functions may be more pronounced (Li and Wang, 2022; Bliemsrieder et al., 2022).

Against this background, electroencephalographic (EEG) Microstate analysis serves as a powerful tool for investigating the dynamic changes in brain function under high-altitude hypoxic conditions. EEG Microstates refer to relatively stable spatial potential distribution patterns that persist for a short duration (typically 80–120 ms) in high temporal resolution EEG signals (Michel and Koenig, 2018). These states reflect the rapid dynamic reorganization of brain functional networks and are regarded as the basic spatiotemporal units of brain information processing (Zerna et al., 2021). By dividing EEG activity into several quasi-stable Microstates, this method facilitates the analysis of the spatiotemporal dynamic characteristics of brain activity (Meng et al., 2023).

Studies have demonstrated that Microstate analysis can reveal changes in Microstates associated with specific cognitive processes, such as facial recognition or semantic judgment, during cognitive tasks (Michel and Koenig, 2018). In high-altitude hypoxic environments, the brain’s response to hypoxic stimulation is characterized by complex and temporally dynamic changes. Therefore, Microstate analysis enables a more precise examination of the brain’s instantaneous dynamics, facilitating the exploration of dynamic changes in specific cortical regions under hypoxic conditions. Through cluster analysis of continuous EEG signals, four classic Microstate patterns with high reproducibility are typically identified, labeled Microstates A, B, C, and D. These have been associated with the auditory network, visual network, default mode network, and attention network, respectively (Wang et al., 2023). Notably, Microstate D corresponds to the prefrontal cortex, parietal lobe, and frontoparietal junction—regions crucial for cognitive control.

A study has shown that acute high-altitude hypoxia at 4,000 m attenuates the stability of Microstates associated with executive control and attention by enhancing slow-wave EEG oscillations, reducing alpha-band power, and flattening the 1/f aperiodic slope. These changes are accompanied by abnormal functional integration in the frontoparietal regions, with Microstate-related alterations converging with characteristics observed in brain aging (Coronel-Oliveros et al., 2024). Another study conducted at 3,650 m found that individuals with poor altitude acclimatization exhibited significantly higher coverage of EEG Microstate D compared to those with good acclimatization, along with abnormally increased transition probabilities between Microstate D and other Microstates (Li et al., 2024). These findings suggest that hypoxia leads to overactivation of Microstate D and disrupts its coordinated regulation with other cognition-related Microstates. Thus, the level of altitude acclimatization serves as a crucial factor influencing the function of the brain’s attention network. Based on these observations, it can be inferred that when individuals experience maladaptation to high altitude (i.e., AAI < 1.7228), abnormalities may emerge in parameters associated with Microstate D.

Previous studies have found that elevated uric acid disrupts the structural and functional integrity of key brain regions—including the prefrontal cortex, thalamus, and basal ganglia—by activating neuroinflammatory pathways, inducing oxidative stress imbalance, and impairing cerebrovascular endothelial function. These effects subsequently compromise the synergistic operation of neural circuits involved in executive control and attentional regulation. Notably, the aforementioned brain regions are closely associated with the attention network corresponding to Microstate D (Lin et al., 2019; Hu et al., 2020; Wang et al., 2024).

In summary, the present study employs EEG Microstate analysis to investigate the neural mechanisms by which individual differences in altitude adaptation—quantified by the altitude adaptation index (AAI)—influence attentional network function through the modulation of serum uric acid (SUA) levels in high-altitude environments. Furthermore, we aim to explore the feasibility of utilizing Microstate D parameters, which are associated with the attention network, as potential neural markers reflecting the impact of AAI on attentional network function in high-altitude populations. The following hypotheses are proposed:

(1) In high-altitude hypoxic environments, a significant negative correlation exists between the altitude adaptation index (AAI) and serum uric acid (SUA) levels. Specifically, lower AAI—indicating poorer altitude adaptation—is associated with higher SUA levels.
(2) In high-altitude hypoxic environments, SUA mediates the effect of AAI on EEG Microstate D parameters. That is, AAI indirectly influences Microstate D-related metricsby modulating SUA levels, thereby supporting the mediating pathway of AAI → SUA → Microstate D.

In recent years, the number of people traveling to and living in high-altitude areas has been steadily increasing, and growing attention has been paid to the physical and mental health of high-altitude populations. Research on high-altitude hypoxia and human health has also been expanding annually, yielding certain achievements. However, studies on the kidney-brain crosstalk function under hypoxic conditions are still in the preliminary exploration stage, and its specific mechanisms as well as the regulatory role of the Altitude Adaptation Index (AAI) have not been fully investigated. This study aims to provide new theoretical evidence for protecting the health of high-altitude populations.

## 2 Materials and methods

### 2.1 Participant recruitment and data collection

A total of 203 participants were randomly recruited from Chengguan District, Lhasa City, Tibet (average altitude: 3,650 m).

This study randomly recruited 203 participants in Chengguan District, Lhasa, Tibet, at an average altitude of 3,650 m. During the study period, the average local temperature was 15.7 °C, the average relative humidity was 55%, and the average atmospheric pressure was 655 hPa. Inclusion criteria: (1) No history of taking drugs relevant to this study; (2) no prior surgery related to the experimental variables; (3) right-handed; (4) no neurological or psychiatric disorders. Exclusion criteria: (1) History of brain injury or substance addiction; (2) diagnosis of kidney-related diseases; (3) recent use of anti-altitude sickness medications.

Some data were excluded to ensure the overall validity and reliability of the dataset due to participant withdrawal during data collection (11 cases), unavailable data due to objective reasons (*n* = 17), and univariate outliers beyond 3 standard deviations (*n* = 2). After applying the aforementioned criteria, the final sample consisted of 173 participants, including 132 females and 41 males (mean age ± standard deviation: 28.03 ± 8.55 years, range: 17–61 years; mean BMI ± standard deviation: 23.07 ± 3.86, range: 16.41–33.56). All participants signed a written informed consent form and were compensated for their participation. This study was approved by the Ethics Committee of Tibet University and conducted in accordance with the Declaration of Helsinki.

### 2.2 Physiological index collection

Physiological indices of participants were collected and recorded by professional physicians using advanced instruments at Tibet Fukang Physical Examination Center. Prior to the measurements performed by physicians, all participants first completed a questionnaire to provide basic demographic information, followed by a physical examination. Serum samples were collected after an overnight fast for complete blood count testing. A total of 3 mL of whole blood was collected using anticoagulant-free vacuum tubes (BD, red cap). After standing for 30 min to coagulate, the samples were centrifuged at 3,000 rpm for 10 min to separate the serum. The serum was aliquoted and stored at −80 °C until detection. Serum uric acid concentration was determined by the uricase-peroxidase method (Roche Cobas 8000, reagent catalog number: 05168787) in strict accordance with the manufacturer’s protocol. Blood samples were obtained via venous sampling for HCT analysis, and SpO₂ was measured using a finger-clip pulse oximeter (YUWELL YX306, Jiangsu Yuwell Medical Equipment & Supply Co., Ltd., Jiangsu, China), with a device output sampling frequency of 1 Hz. Prior to the experimental assessment, participants were required to rest for at least 30 min upon arrival at the laboratory. During the measurement, participants maintained a comfortable seated position with their right hand placed on the table in front of them, both hands resting naturally on the table with wrists and palms facing downward, avoiding any limb movement. The oximeter was attached to the participant’s right index finger (Sun, 2010). A total of three measurements were taken during the entire experimental assessment phase, with a 30-s interval between each measurement. The average value was calculated as the analytical indicator. The Altitude Adaptation Index (AAI) was calculated as the ratio of SpO₂ to HCT (AAI = SpO₂/HCT).

### 2.3 EEG data collection and preprocessing

Electroencephalographic (EEG) data were collected using the ANT Neuro 64-electrode system^1^ at a sampling rate of 1,000 Hz. Electrodes were placed following the standard 10–20 system, with all impedances maintained below 5 kΩ. The online reference electrode was positioned at CPz, and the ground electrode at FCz. The amplifier amplified the signals to facilitate continuous EEG recording. Online filtering was performed within a bandwidth of 0.01 to 100 Hz, with data collected for 5 min under eyes-closed resting state. Data processing and analysis were conducted using the EEGLAB toolbox (Delorme and Makeig, 2004) and Fieldtrip (Oostenveld et al., 2011) in MATLAB (version 2021b, The MathWorks). Prior to processing, the raw data were downsampled to a quarter of the original sampling rate (256 Hz).

For offline processing of EEG data, the open-source toolbox EEGLAB (Delorme and Makeig, 2004) in MATLAB (version 2021b, The MathWorks, Inc., Natick, MA, USA) was used. Continuous EEG data *were subjected to a 2 Hz high*-pass filter, and data below 20 Hz were low-pass filtered using a basic finite impulse response (FIR) filter. After identifying potential bad electrodes, the data were corrected via spherical interpolation. Subsequently, continuous 0.5-s time segments were extracted, and contaminated segments were manually marked and discarded.

Independent Component Analysis (ICA) algorithm (Jung et al., 2000) was used to correct EEG data contaminated by muscle, heart, line noise, and channel noise. The ICLabel plugin (Pion-Tonachini et al., 2019) was applied to remove components with a probability exceeding 0.7 for electromyography, electrocardiography, line noise, and channel noise (Bagdasarov et al., 2022) (an average of 5 components were removed per participant, with a standard deviation of 2). Time segments with amplitudes exceeding ±100 μV were excluded. Subsequently, artifact-free time segments were concatenated into continuous EEG data for Microstate analysis. Finally, the EEG data were re-referenced to the average reference, completing the data processing sequence.

### 2.4 Microstate analysis

Microstate analysis of resting-state electroencephalographic (rs-EEG) data was performed using the Microstate Toolbox (Poulsen et al., 2018). First, the preprocessed EEG data were band-pass filtered at 2–20 Hz to remove low-frequency noise (e.g., eye movements and blinks) and high-frequency noise (e.g., muscle activity) (Li et al., 2022; Seitzman et al., 2017). Subsequently, for each participant in each group, the Global Field Power (GFP) was calculated as the standard deviation of EEG signals across all electrodes at each time point, representing the intensity of instantaneous EEG activity over the scalp. Since EEG scalp topographies remain stable around GFP peaks, only the EEG topographies corresponding to the peaks of the GFP waveform were extracted and submitted to the clustering algorithm. We employed a modified *k*-means clustering algorithm to obtain topographies based on topographic similarity while ignoring polarity. This modified *k*-means clustering algorithm was applied multiple times with the number of clusters (*k*) ranging from 2 to 8, resulting in 2–8 topographies for each participant and each group. Next, a second topographic clustering was performed at the group level using individual topographies from different participants. Similarly, polarity was ignored in this process. The optimal number of clusters (4 in this study) was determined via cross-validation, and the topographies of both groups were clustered into 4 Microstates, respectively. Finally, using the 4 Microstate categories obtained at the group level as template maps, the scalp potential topography of each participant at each time point was back-fitted to one of the 4 categories (A–D) based on the spatial correlation between the template maps and the participant’s scalp potential topographies.

After obtaining participants’ Microstates, three commonly used EEG Microstate features—Duration, Occurrence, and Coverage—were extracted from the Microstate time series for subsequent analysis. Duration refers to the average length of time a given Microstate category is detected in the EEG data, reflecting the stability of the underlying neural configuration. Occurrence represents the frequency at which a given Microstate remains dominant (number of occurrences per second), indicating the characterization trend of underlying neural activation; Coverage is the ratio of the total time a given Microstate remains dominant to the total recording time; unlike static metrics such as duration and coverage, Microstate transition probabilities directly characterize the rapid switching patterns of brain networks and reflect the propensity for sequential activation of distinct large-scale brain networks.

### 2.5 Statistical analysis

Data analysis was performed using SPSS 27.0 to describe the demographic characteristics and physiological indices of participants. Pearson correlation analysis was used to assess the strength of linear associations between AAI, serum uric acid, and EEG Microstate data, with a significance level set at *p* < 0.05. To control the inflation of Type I error rate caused by multiple comparisons, the *p*-values obtained from the correlation analysis were corrected by FDR (False Discovery Rate). The significance level after correction was still set as *p* < 0.05. Participants were divided into high and low uric acid groups according to the criteria in *Chinese Guidelines for the Diagnosis and Treatment of Hyperuricemia and Gout (2019)* (Feng et al., 2020) (“using > 420 μmol/L as the gender-neutral diagnostic criterion for hyperuricemia”), and into well-adapted and poorly adapted groups based on AAI scores. Normality tests were conducted on parameters associated with Microstate D. Independent samples *t*-tests were used for variables conforming to normal distribution, while non-parametric tests were applied for those not conforming to normal distribution, to compare differences in Microstate D indices between groups. Drawing on previous theoretical frameworks and the basic principles of this study, SUA was introduced as a mediating variable in the neural mechanism underlying the impact of different AAI levels on Microstate D-related indices. Mediation effect analysis was performed using the PROCESS 4.2 plugin, with validation via the Bootstrap method to enhance result robustness. This method provides the empirical distribution of the mediation effect and its 95% confidence interval; a confidence interval that does not contain 0 indicates a significant mediation effect.

## 3 Results

### 3.1 Microstate topographies

**Figure 1:**
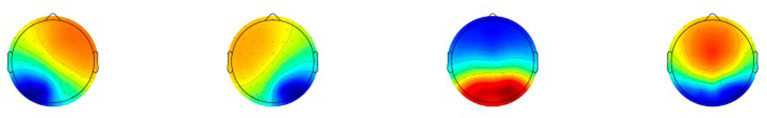
Topographies of the four classic EEG microstates. The figure shows the four stable Microstate topographies (A–D) extracted from resting-state EEG data via cluster analysis. The spatial distributions from left to right correspond to Microstate A,B,C, and D, respectively.

Typical Microstate topographies in resting-state EEG data were extracted via cluster analysis (Figure 1), with a total of 4 stable patterns (A–D) identified. The spatial distribution of each topography was consistent with classic Microstate studies: Microstate A exhibited an oblique gradient from the left prefrontal lobe to the right posterior parietal lobe; Microstate B showed a symmetric frontopolar-occipital distribution across the left and right hemispheres; Microstate C presented a frontocentral-posterior temporal distribution pattern; Microstate D displayed an oblique distribution from the right prefrontal lobe to the left posterior parietal lobe.

### 3.2 Correlations among SUA, AAI, and Microstate B indices

This study examined the correlations among AAI, SUA, and Microstate D-related parameters (Coverage D, Duration D, Occurrence D, TPDA, TPDB, TPDC, TPAD, TPBD, TPCD) in this study (Table 1). The results revealed a series of significant correlations: AAI was negatively correlated with SUA (*r* = −0.460, *p* < 0.01); AAI was positively correlated with TPBD (*r* = 0.193, *p* < 0.05) and negatively correlated with TPDC (*r* = −0.156, *p* < 0.05); SUA was positively correlated with TPDA (*r* = 0.167, *p* < 0.05) and TPDC (*r* = 0.203, *p* < 0.01). However, after FDR correction for multiple comparisons, only the negative correlation between AAI and SUA remained significant, while the correlations between AAI and TPBD, and between SUA and TPDC, became marginally significant. This suggests that the relationships among AAI, SUA, and Microstate D-related parameters may be relatively weak or insufficiently powered to survive strict multiple comparison control given the current sample size. Nevertheless, subsequent mediation analysis revealed significant indirect effects via SUA, indicating that meaningful relational structures among variables may still exist even in the absence of strong bivariate correlations.

**Table 1:** Correlations.

	AAI	SUA	Coverage D	Duration D	Occurence D	TPDA	TPDB	TPDC	TPAD	TPBD	TPCD
AAI	1	−0.460^**^	−0.040	−0.030	−0.032	−0.420	0.036	−0.125	0.005	0.193^*^	−0.156^*^
SUA	−0.46^**^	1	0.129	0.112	0.094	0.167^*^	0.073	0.203^**^	0.017	−0.104	0.069

^*^p<0.05, ^**^p<0.01, ^***^p<0.001.

### 3.3 SUA differences: adaptive vs. maladaptive groups

Based on the AAI, participants were divided into an adaptive group (A, *n* = 147; AAI = 2.079 ± 0.23) and a maladaptive group (M, *n* = 26; AAI = 1.61 ± 0.12). An independent samples *t*-test comparing SUA levels between the two AAI groups revealed a statistically significant difference (*t* = −5.107, *p* < 0.001). Specifically, the high SUA group (*n* = 28; SUA = 520.89 ± 66.34 μmol/L) consisted of 18 males and 10 females, while the low SUA group (*n* = 145; SUA = 298.90 ± 58.46 μmol/L) included 23 males and 122 females. The independent samples *t*-test for SUA confirmed a significant difference (*t* = −5.107, *p* < 0.001), indicating that individuals with poor altitude adaptation exhibited significantly higher uric acid levels (Table 2).

**Table 2:** Differences in SUA among different AAI levels.

	AAI	*N*	*M* ± *SD*	*t*	*p*
SUA	A	147	319.37 (90.987)	−5.107^***^	*p* < 0.001
	M	26	422.27 (114)		

^*^p<0.05, ^**^p<0.01, ^***^p<0.001. A = Adaptive group; M = Maladaptive group.

### 3.4 Microstate differences: adaptive vs. maladaptive groups

Non-parametric tests were conducted to compare Microstate-related parameters (TPDC, TPDA, TPBD, TPCD) between groups with different AAI levels. The results showed no significant between-group differences for TPDC (*Z* = −0.754, *p* = 0.451) or TPDA (*Z* = −0.045, *p* = 0.964). However, statistically significant differences were observed for TPBD (*Z* = −2.088, *p* = 0.037) and TPCD (*Z* = −2.406, *p* = 0.016) (Table 3).

**Table 3:** Nonparametric test (different AAI levels).

	AAI	*N*	Q1上四分位数	M中位数	Q3下四分位数	*Z*	*p*
Transition Probability D→C	A	147	0.283	0.337	0.405	−0.754	0.451
	M	26	0.297	0.338	0.478		
Transition Probability D→A	A	147	0.266	0.297	0.353	−0.045	0.964
	M	26	0.255	0.303	0.347		
Transition Probability B→D	A	147	0.241	0.292	0.341	−2.088^*^	0.037
	M	26	0.219	0.267	0.303		
Transition Probability C→D	A	147	0.338	0.376	0.437	−2.406^*^	0.016
	M	26	0.338	0.440	0.530		

^*^p<0.05, ^**^p<0.01, ^***^p<0.001. A = Adaptive group; M = Maladaptive group.

### 3.5 Mediation of SUA between AAI and Microstate D

**Figure 2:**
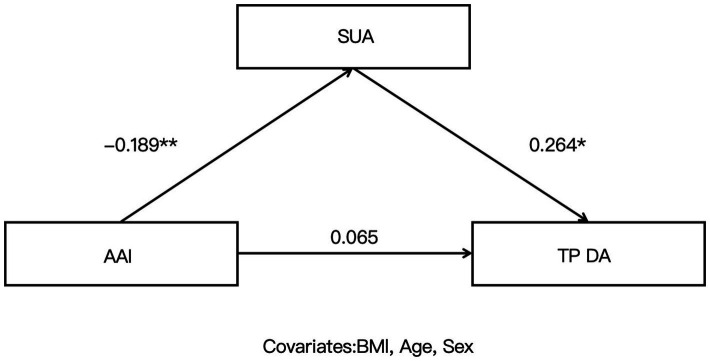
Mediation role of serum uric acid (SUA) in the relationship between altitude acclimatization/adaptation index (AAI) and the transition probability from Microstate D to Microstate C (TPDC).

**Figure 3:**
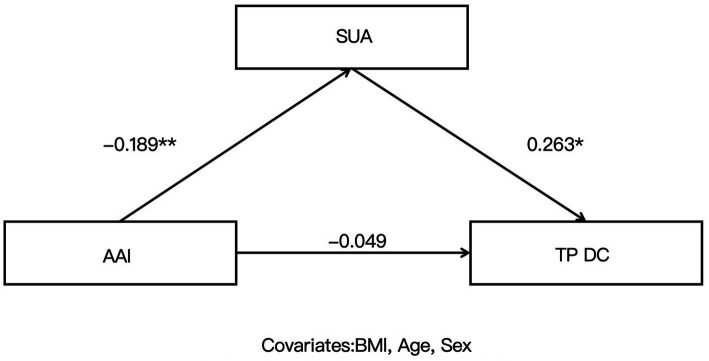
Mediation role of serum uric acid (SUA) in the relationship between altitude acclimatization/adaptation index (AAI) and the transition probability from Microstate D to Microstate C (TPDC).

Given that age, sex, and BMI may influence SUA and HCT levels, these variables were included as covariates in the mediation analysis. Mediation analyses were conducted with AAI as the independent variable, SUA as the mediator, and Microstate-related parameters (TPDA, TPDC, TPBD, TPCD) as dependent variables to examine the mediating role of SUA in the relationship between AAI and each Microstate parameter. The results were as follows: the indirect effect of the TPDA–SUA–AAI pathway was significant (*β* = −0.050, SE = 0.027, 95% CI [−0.112, −0.007]); the indirect effect of the TPDC–SUA–AAI pathway was also significant (*β* = −0.050, SE = 0.032, 95% CI [−0.123, −0.001]). However, the indirect effects were not significant for the TPBD–SUA–AAI pathway (*β* = 0.005, SE = 0.020, 95% CI [−0.034, 0.047]) or the TPCD–SUA–AAI pathway (*β* = 0, SE = 0.023, 95% CI [−0.050, 0.044]) (Tables 4–7 and Figures 2, 3).

**Table 4:** Mediation analysis of TPDA-SUA-AAI.

	*β*	se	95%CI	Relative effect size
Total effect	0.015	0.097	[−0.176, 0.206]	—
Direct effect	0.065	0.097	[−0.127, 0.258]	424.18%
Indirect effect	−0.050^*^	0.027	[−0.112,-0.007]	−324.18%

*p<0.05.**p<0.01.***p<0.0011.

**Table 5:** Mediation analysis of TPDC-SUA-AAI.

	*β*	se	95%CI	Relative effect size
Total effect	−0.099	0.096	[−0.288，0.090]	—
Direct effect	−0.049	0.097	[−0.240，0.141]	49.8%
Indirect effect	−0.050^*^	0.032	[−0.123，-0.001]	50.2%

*p<0.05.**p<0.01.***p<0.0011.

**Table 6:** Mediation analysis of TPBD-SUA-AAI.

	*β*	se	95%CI	Relative effect size
Total effect	0.162	0.096	[−0.028, 0.352]	—
Direct effect	0.157	0.099	[−0.038, 0.352]	96.7%
Indirect effect	0.005	0.020	[−0.034, 0.047]	3.3%

**Table 7:** Mediation analysis of TPCD-SUA-AAI.

	*β*	se	95%CI	Relative effect size
Total effect	−0.181	0.097	[−0.372, 0.010]	—
Direct effect	−0.182	0.099	[−0.378, 0.015]	100.28%
Indirect effect	0	0.023	[−0.050, 0.044]	−0.28%

## 4 Discussion

The present study, involving 173 high-altitude residents in Lhasa, examined the relationships among AAI, SUA, and Microstate D parameters. The findings validated the mediating pathway of “AAI → SUA → Microstate D,” revealing the neural mechanism by which AAI influences the dynamic regulatory capacity of the brain’s cognitive control network under high-altitude hypoxic conditions.

The results showed that SUA had a significant negative predictive effect on AAI, with extremely significant differences in AAI levels between the high SUA group and the low SUA group. This indicates that elevated SUA levels significantly reduce individuals’ high-altitude adaptation capacity. This mechanism may be related to the increase in blood viscosity caused by elevated SUA levels, as well as oxidative stress and inflammatory responses induced by long-term elevated SUA levels, which damage vascular endothelium and renal function, thereby affecting the high-altitude adaptation process (Meng et al., 2025; Gao et al., 2023). Additionally, studies have found that in populations with long-term high-altitude residence, hyperuricemia is closely associated with high-altitude polycythemia (HAPC), and HAPC is one of the important manifestations of chronic mountain sickness (Chen et al., 2025) suggesting that hyperuricemia may be a marker of poor high-altitude adaptation.

After grouping participants based on the AAI cutoff value, selective differences in Microstate D transition probabilities were observed between the adaptive and maladaptive groups. Significant between-group differences were found for TPBD and TPCD, whereas no significant differences were observed for TPDC or TPDA. These findings suggest that a lower AAI reflects reduced altitude acclimatization capacity, wherein insufficient blood oxygen supply fails to meet the high energy demands of the prefrontal-parietal regions. This impairs the efficiency of input information exchange between the cognitive control network and both the visual and default mode networks, manifested as abnormal transition probabilities from other Microstates to Microstate D. The absence of significant group differences in TPDC and TPDA may be attributed to their role in reflecting output transitions from the cognitive control network. These processes are not only influenced by blood oxygen supply but also depend more heavily on intrinsic neural activation rhythms and cross-network synaptic transmission efficiency. Being less directly modulated by hypoxic stress, TPDC and TPDA may better reflect the baseline functional properties of the cognitive control network and remain relatively stable under acute hypoxic exposure. This suggests that TPDC and TPDA could serve as potential indicators for assessing long-term altitude adaptation of the cognitive control network. Consistent with previous studies, these findings confirm that hypoxia impairs the efficiency of information integration between the cognitive control network and other brain networks, further supporting AAI as a key metric for evaluating the dynamic interactive function of the cognitive control network at high altitudes (Hofmeijer et al., 2014).

The core finding of this study is that SUA mediates the relationship between AAI and specific Microstate D transition probabilities. Specifically, AAI influenced TPDA and TPDC indirectly through SUA, with significant indirect effects, whereas no such indirect effects were observed for TPBD or TPCD. This indicates that AAI does not modulate all Microstate D transition probabilities via SUA uniformly, but rather exhibits a targeted regulatory pattern. The present study extends this mechanism in a healthy population at 3,650 m, demonstrating that under high-altitude hypoxic conditions, AAI’s regulation of cognitive control network-related Microstate transitions relies on SUA mediation only for output transitions from D to A and D to C. In contrast, input transitions from B to D and C to D are not mediated by SUA. These findings suggest that reduced AAI at high altitude first elevates SUA levels, which in turn leads to abnormalities in transition probabilities from Microstate D to A and C, whereas the impact of AAI on input transitions to Microstate D is mediated through other pathways independent of SUA metabolic regulation.

This finding further delineates the specific scope within which SUA mediates the regulation of cognitive control network function by AAI under high-altitude hypoxic conditions. It aligns with previous research indicating that elevated uric acid levels are associated with cognitive decline in attention and executive function (Tang et al., 2022), while also resonating with evidence suggesting that uric acid may confer certain cognitive benefits through its antioxidant properties (Mijailovic et al., 2022). Together, these complementary perspectives provide crucial mechanistic support for the targeted mediating role observed in this study. Moreover, this finding points to a clear direction for future research, namely the need to further investigate the specific roles of factors such as blood oxygen supply, cerebral hemodynamics, or non-SUA-mediated neuroinflammation in AAI’s regulation of input transitions to Microstate D. From a practical perspective, these results offer actionable insights for the assessment and intervention of cognitive control network function in high-altitude populations. Clinically, SUA may be combined with TPDA and TPDC as specific indicators for monitoring the impact of AAI on the output function of the cognitive control network. In contrast, abnormalities in input transitions to Microstate D may require the identification of intervention targets beyond SUA.

In recent years, exploration of the “kidney-brain axis” in high-altitude health research remains in its early stages. Previous studies have primarily focused on the direct association between AAI and brain damage (Hu et al., 2021; Hou, 2019; Zhang et al., 2015; Steinbach & Harshman, 2022), yet the underlying mediating mechanisms have remained unclear. The present study, through mediation analysis, confirms the central role of SUA and, for the first time, establishes a sequential pathway of “systemic adaptation capacity → renal metabolic indicator → brain function (attention network)” under high-altitude hypoxic conditions. These findings provide novel empirical evidence for kidney-brain axis research and fill a critical gap in understanding how altitude acclimatization capacity, via metabolic pathways, mediates the dynamic function of cognitive control networks at high altitude. Furthermore, by integrating AAI, SUA, TPDA, and TPDC, this study offers a quantitative indicator combination with high temporal resolution for assessing cognitive control network function. Compared with traditional cognitive behavioral tests, this combination of indicators enables more sensitive detection of transient interaction abnormalities in the cognitive control network under high-altitude hypoxic stress, thereby providing a novel methodological reference for future research on high-altitude brain function.

As an increasing number of individuals travel to or reside in high-altitude regions, cognitive impairment resulting from maladaptation to high altitude has emerged as a potential health risk. The findings of this study offer actionable targets for clinical intervention: AAI can serve as a core assessment indicator, which, in combination with SUA, TPDA, and TPDC, can be used to establish an early screening system for cognitive control network dysfunction in high-altitude populations. By monitoring changes in AAI levels, it may be possible to predict the risk of SUA elevation and subsequent impairment of cognitive control network output function, thereby enabling early identification of both poor altitude acclimatization and brain functional abnormalities. For individuals with AAI < 1.7228, SUA levels should be closely monitored to facilitate early detection of maladaptation risk. Furthermore, interventions targeting the core independent variable—improving altitude acclimatization capacity (AAI)—may regulate SUA levels at the source and thereby protect cognitive control network function. Strategies such as staged ascent and hypoxic preconditioning training can enhance the body’s acclimatization capacity by improving the balance between blood oxygen supply and hematocrit. Concurrently, dietary adjustments (e.g., reducing intake of high-purine foods) and promoting uric acid excretion through adequate hydration may help lower SUA levels (Song et al., 2025; Jakše et al., 2019; Du et al., 2024), thereby mitigating abnormalities in transition probabilities from Microstate D to A and C mediated by reduced AAI, ultimately preserving the cross-network interactive function of the cognitive control network in high-altitude populations. Moreover, AAI (SpO₂/HCT) is simple and convenient to measure, making it suitable for implementation in primary care settings at high altitudes, and can serve as an important reference indicator for protecting attentional function.

## 5 Limitations

Through rigorous statistical analysis, this study demonstrates that under hypoxic conditions at 3,650 m, SUA serves as a key mediating variable through which AAI regulates output transitions of cognitive control network-related Microstate D. Reduced AAI significantly elevates SUA levels, which in turn mediates abnormalities in transition probabilities from Microstate D to A and C, resulting in impaired dynamic regulatory capacity of the cross-hemispheric cognitive control network. This finding provides important empirical evidence and mechanistic insights into the complex “acclimatization–metabolism–brain function” relationship at high altitude. Clinically, it identifies actionable “screening–intervention” targets; from a public health perspective, it offers a scientific basis for preventing and managing cognitive health risks in high-altitude populations. Furthermore, it provides new directions for subsequent research on the “kidney–brain axis” in high-altitude medicine, holding significant academic value and practical applicability.

However, it should be noted that this study has limitations that warrant consideration: (1) The sample was recruited from the Chengguan District of Lhasa, which may introduce regional and gender biases. Future studies should expand the sample size and conduct multicenter investigations to validate the generalizability of the findings. (2) The cross-sectional design employed here only revealed associations and mediating relationships among AAI, SUA, and Microstate D parameters, without establishing causal or temporal sequences among these variables. Furthermore, this design could not capture the dynamic evolution of these indicators during the altitude acclimatization process, making it difficult to elucidate the long-term characteristics of AAI regulation of SUA and brain network function under high-altitude conditions. (3) The impact of high-altitude hypoxia on cognitive function is multidimensional (e.g., attentional deficits, memory impairment). Future research should extend the analytical scope to include other Microstates (A, B, and C, associated with the auditory network, visual network, and default mode network, respectively) to explore the mechanisms linking SUA with other cognitive networks.

## Data Availability

The original contributions presented in the study are included in the article/supplementary material, further inquiries can be directed to the corresponding author.

## Footnotes

1
https://www.ant-neuro.com
